# Clear aligners’ effects on aesthetics: evaluation of facial wrinkles

**DOI:** 10.4317/jced.54925

**Published:** 2018-07-01

**Authors:** Romeo Patini, Patrizia Gallenzi, Simonetta Meuli, Valeria Paoloni, Massimo Cordaro

**Affiliations:** 1 PhD, DDS. Institute of Dentistry, School of dentistry, Catholic University of Sacred Heart, Largo A. Gemelli, 1 - 00168 Rome, Italy; 2 DDS. Institute of Dentistry, School of dentistry, Catholic University of Sacred Heart, Largo A. Gemelli, 1 - 00168 Rome, Italy; 3 DDS. Clinical Sciences and Translational Medicine, University of Rome “Tor Vergata”, Viale Oxford, 81 – 00133 Rome, Italy; 4 MD, DDS. Institute of Dentistry, School of dentistry, Catholic University of Sacred Heart, Largo A. Gemelli, 1 - 00168 Rome, Italy

## Abstract

**Background:**

To evaluate the facial aesthetic effects of orthodontic treatment performed with clear aligners and to compare it to an untreated control group, on lower third facial ageing in adult patients through the use of the Wrinkle Severity Rating Scale (WSRS) at the beginning (T0) and at the end (T1) of the study period.

**Material and Methods:**

A clear aligners treated group (TG) of 68 patients was compared to a control group of 33 untreated patients (UG). Each group was divided in 2 subgroups according to age: subgroup 1 if under 40 years of age and subgroup 2 if over. Facial aesthetics of the lower third was evaluated at T0 and T1 by a panel of five aesthetic experts with WSRS.

**Results:**

Statistically significant changes were found in all subgroups comparing WSRS scores at T0 and T1. The between group comparisons revealed that wearing clear aligners produces a statistically relevant improvement in lower third facial aesthetics both in younger (*p*< 0.05) and older (*p*< 0.001) patients.

**Conclusions:**

The present retrospective cohort study has successfully shown that malocclusion therapy conducted through the use of clear aligners in a population of adults affected by dental crowding has beneficial effects on lower third facial ageing.

** Key words:**Orthodontics, clear aligners, facial aesthetics, facial wrinkles, compliance.

## Introduction

Ageing is an inevitable life process: from birth human genes lead the body through a series of changes ranging from growth and development to maturation, ageing and finally death. Facial ageing is a complex physiological process, involving hard and soft tissues, essentially characterized by loss of soft tissue muscle tone and of hard tissue volume.

In the dentate patient the facial ageing is believed to be totally due to soft tissue changes. Ageing of the skin is associated with progressive atrophy of the dermis as well as changes in the dermal tissues. This leads to thinning, wrinkling, sagging and laxity of the facial skin. Moreover the muscles of the head and neck show a significant reduction in cross-sectional area and density with age (1).

The increased interest in aesthetics procedures has required the development of scales to measure the degree of ageing, the severity of facial wrinkles and the level of improvement resulting from such procedures. Numerous different instruments have been proposed with the aim of creating a standardized tool for the measurement of ageing such as questionnaires or the 3-Dimensional photogrammetry (2). Validated scales could be also useful for assessing patient-reported outcomes, such as quality of life and satisfaction with treatment. One of the above mentioned scales is a photograph-based five-point scale, named Wrinkle Severity Rating Scale (WSRS), that is designed for quantifying facial folds from 1 = absent to 5 = extreme (3).

Orthodontics and orthognathic surgery, with only a moderately increased risk rate of complications (4) and a more positive effect on patient’s compliance (5), can help give facial harmony, where malocclusions created a situation of abnormalities in bone bases, teeth position, muscle function and posture of lips and tongue. The consequence of a correct orthodontic treatment is the finding of a face harmony, highlighted with the face aesthetic analysis (6).

Clear aligners consist in a series of transparent aligners that are able to perform orthodontic movements without compromising the aesthetic of the smile. A recent systematic review demonstrated that clear aligner treatment is effective in aligning and levelling the arches in non-growing subjects (7).

The aim of this retrospective cohort study was to evaluate the effects of orthodontic treatment performed with clear aligners on lower third facial ageing in adult patients through the use of WSRS by a panel of five aesthetic experts.

## Material and Methods

-Subjects

The examined sample consisted of subjects enrolled in a retrospective observational study at the dental clinic of “A. Gemelli” Foundation – Teaching Hospital in Rome. The study project was approved by the Ethical Committee at the Catholic University of Sacred Heart in Rome. The following inclusion criteria had to be fulfilled by the subjects enrolled in the study:

• Skeletal class I (ANB: 2°+/- 2°);

• Overjet between 0 and 4 mm;

• Overbite between 0 and 4 mm;

• Molar and canine class I;

• Moderate upper and lower crowding (with dento-basal discrepancy from 4.1 to 7.0 mm);

• Presence of all permanent teeth except for third molars.

The following exclusion criteria were established:

• Previous orthodontic treatment;

• Surgical interventions or aesthetic treatments in head and neck district;

• Loss or gain more than 2 kg during the study period.

A sample of 101 patients with moderate crowding was enrolled for the study, and informed consent was obtained. The following records were collected in the study sample at the initial observation (T0) and after an average period of 12 months (T1): intraoral and extraoral photographs, orthodontic casts, latero-lateral teleradiography and orthopanoramic radiography.

All subjects, homogeneous for dental and skeletal relationship, were assigned to one of the following two groups:

• Treatment of dental crowding with a set of clear aligners (TG);

• Patients that, for certain personal motivations, decided to defer orthodontic treatment (UG).

Each group was further divided according to age into subgroups: subgroup 1 if under 40 years of age and subgroup 2 if over.

Participants enrolled in this study showed the following distribution:

• TG1: 39 subjects, mean age of 33.31 years at T0, 20 female and 19 male;

• TG2: 29 subjects, mean age of 45.52 years at T0, 15 female and 14 male;

• UG1: 20 subjects, mean age of 31.42 years at T0, 10 female and 10 male;

• UG2: 13 subjects, mean age of 46.54 years at T0, 7 female and 6 male.

Demographics of the above mentioned group and statistical comparisons regarding age at T0 and study period are shown in  Table 1 and  Table 2.

**Table 1:**
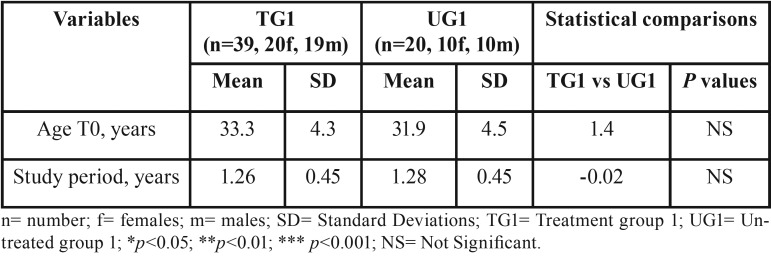
Demographics of the groups TG1 and UG1, study period duration and statistical comparisons.

**Table 2:**
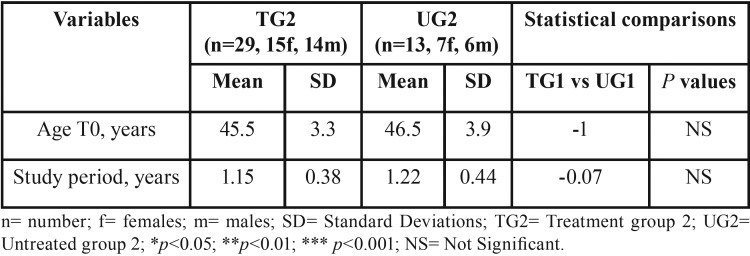
Demographics of the groups TG2 and UG2, study period duration and statistical comparisons.

-Treatment protocol

In the TG a set of clear aligners (Invisalign®, Align Technology, San José, CA, USA) was used as the sole appliance. Each aligner has a thickness of about 0.75 mm. Patients were instructed to wear the aligners for 22 hours/day, to remove them only for meals and dental cleaning and to regularly change them every 14 days. Patients belonging to the UG did not receive any form of orthodontic treatment during the study period.

Extraoral frontal projections photographs at T0 and T1 were taken in the same room with steady light conditions with the same camera parameters (distance, focus and flash). Photographs were taken on a wide screen with cutaneous Frankfurt plane parallel to the floor; male patients were kindly asked to have their face shaved and female patients to have their make-up removed before taking pictures. All patients belonging to the treated groups had their clear aligner removed before taking the photographs.

-Facial ageing assessment

The evaluation of facial folds of the lower third was done through the WSRS.

A panel of five aesthetic experts (a plastic surgeon, a dermatologist, an aesthetic physician and two orthodontists) previously calibrated (Cohen’s Kappa = 0.893) were asked to express a judgement regarding the lower third facial ageing of patients from 1 = absent to 5 = extreme. Pictures from T0 and T1 were randomly allocated through the use of a computer generated code and shown to the aesthetic experts in a portable document format (pdf) file.

The evaluations and statistical analysis were carried out double blind since each photograph was matched with a code, known by an author not involved in the clinical and statistical evaluations.

-Statistical methods

Due to the lack of scientific reports regarding the evaluation of the impact of orthodontic therapy on facial ageing, the sample size calculation of each group was conducted by performing a power analysis based on the results of a previous pilot study, to detect at least a 1 grade difference between groups, with a standard deviation of 0.9 grade, an alpha value of 0.05 and a power of 0.8 and was calculated to be 13 subjects.

To determine the reliability of the method, WSRS measurements on half of the subjects were performed by one trained examiner and repeated after an interval of approximately two weeks. A paired t-test was used to compare the two measurements (systematic error). The magnitude of the random error was calculated by using the method of moment’s estimator (MME). Exploratory statistics revealed that all variables were normally distributed (Kolmogorov-Smirnov test) with equality of variances (Levene’s test). Lower third facial wrinkle changes during study period (T1-T0) were evaluated and paired t-tests were used to assess statistical significance within each group. A comparison of changes between the two groups was also carried out using unpaired t-tests. All comparisons were considered significant at *p* < 0.05. All statistical analyses were performed using the Statistical Package for Social Sciences version 15.0 (SPSS Inc., Chicago, Illinois, USA).

## Results

The present investigation reached an adequate power (> 0.85) because of the number of subjects enrolled in the examined groups.

The study period lasted 1.23 years (SD: 0.43 years). No systematic error was found between the WSRS repeated measurements. For the lower third facial wrinkle changes the random error was 0.01 marks. No significant between-group differences were found for age of the participants at T0 and for the study period duration ( Table 1, Table 2).

Skeletal, molar and canine class, overjet and overbite did not register any significant change during the study period.

Regarding subgroups 1 the overall aesthetics of the lower third of the face showed a statistically significant change of -0.21 (*p* < 0.05) in the TG and of 0.27 (*p* < 0.05) in the UG. Consequently the difference between TG1 and UG1 was -0.29 (*p* < 0.05).

Regarding subgroups 2 the overall aesthetics of the lower third of the face showed a statistically significant change of -0.28 (*p* < 0.01) in the TG and of 0.30 (*p* < 0.05) for the UG. Thus the difference between TG2 and UG2 was -0.65 (*p* < 0.001) ( Table 3, Table 4).

**Table 3:**
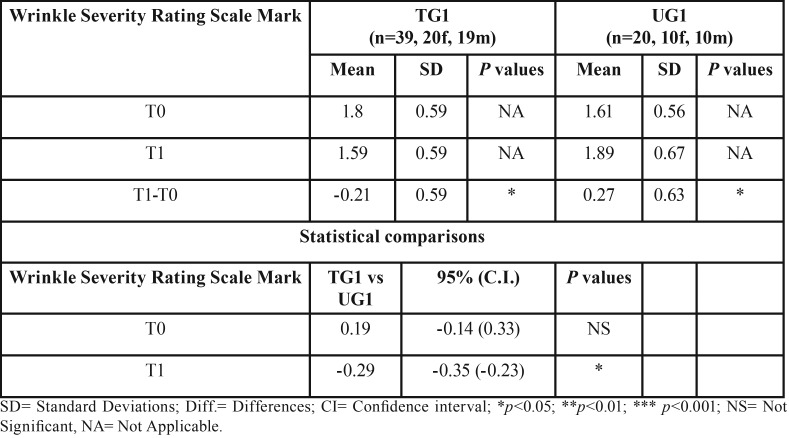
Descriptive statistics and statistical comparisons of the WSRS marks at T0 and T1 in subgroups TG1 and UG1.

**Table 4:**
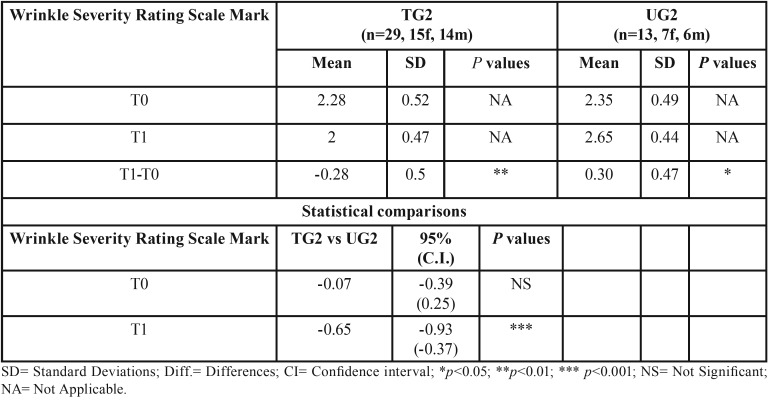
Descriptive statistics and statistical comparisons of the WSRS marks at T0 and T1 in subgroups TG2 and UG2.

## Discussion

This retrospective cohort-study, whose aim was to evaluate the effects of orthodontic treatment on lower third facial ageing in adult patients, has successfully shown that wearing clear aligners during orthodontic treatment has beneficial effects on face soft tissues when compared to the aesthetic impact of patients of an untreated group.

The results found in the present study show the presence of an additional effect on the lower third facial aesthetics in patients undergoing orthodontic therapy with clear aligners. To the best of our knowledge no other report had previously highlighted this particular effect of orthodontic therapy; for this reason any comparison with the previous scientific literature is fairly inaccurate to do.

A possible explanation of this finding is that the mechanism of dental aligners could be compared to the effects of splints that interact with the vertical intraoral dimension. In fact wearing two aligners (one for each arch), with a thickness of about 0.75 mm each, could lead to an increasing of the intraoral vertical dimension, though it is limited to the time of the aligners application. Constantly wearing the aligners, a slight modification of the vertical dimension could have beneficial effect also in the intra-articular compression, even with a repositioning of the joint (8). Positive effects are also reported in patients affected by obstructive sleep apnoea syndrome whose diagnosis is even more complex in the event of a decrease of the vertical dimension (9).

Such beneficial effects are also reported by Okeson that demostrated that occlusal splints can improve occlusal conditions, reducing muscular activity (10).

The result of this splint effect, due to the wearing of the aligners for 22/24 hours a day for at least one year, has been evaluated on the lower third face soft tissues and considered as an anti-aging effect. Such consideration is shown by the statistical analysis resulting from the evaluations of 5 facial aesthetics experts.

The second possible mechanism of action of anti-aging effect of clear aligners could be linked to the fact that wearing the aligners involves a number of changes in the cellular and molecular metabolism of bone and muscle structures. In particular, authors reported an improvement related to the whole range of facial features, not just the lower face, using appliances (acrylic pivots), for six months. The mechanisms by which patients’ signs of facial aging are reversed are based on the fact that both muscles and bone respond to mechanical signals. Stretch of the muscle fibres leads to an increase in the bone density due to the activity of the osteocytes and induces release of mechano-growth-factor (MGF) and insulin growth factor 1 (IGF-1) (11).

These two mechanisms of action, mentioned as possible explanations of this anti-aging effect during orthodontic therapy conducted with Invisalign® on adults, could be considered as phenomena that can take place in combination rather than individually.

A statistically significant improvement in facial aesthetics was found in both subgroups of patients but such finding is much more significant in older patients (subgroup 2) (*p* <0.001).

This finding can be explained by suggesting that at older ages, aging mechanisms become more apparent and any aesthetic improvement, although of a minimal clinical significance, can be better noticed as compared to those that may occur at a younger age in which to show the same aesthetic improvement it is required to make a substantial change in the facial soft tissues architecture.

The findings of this study, however, should be interpreted with caution because it has some limitations. Firstly: although the results were statistically significant the size of the sample is quite small. Another limitation is that the judgements given by the experts were expressed looking at photographs: this could unavoidably lead to a partial perception of the whole patient’s facial expressions. In fact it is important to consider that in most cases the facial beauty and self perception is linked to a global face evaluation rather than the appraisal of the depth of wrinkles of a specific part of it. This statement finds support in the fact that in pictures taken at T1 phase patients very often showed a change of their hairstyle or make-up, even in cases where experts’ judgments had revealed a decrease in facial aesthetics, so that patients were asked to remove their make-up before taking all the photographs not to influence the aesthetic evaluations. Probably the biggest limitation of this study is the lack of a control group treated with traditional orthodontic therapy. Such a lack is justified by the fact that the second photograph records (T1) were taken at an average period of 12 months after the beginning of therapy with clear aligners. This moment does not always correspond to the end of therapy. Therefore photographing subjects wearing brackets would have inevitably caused a methodological bias as the circum-oral soft tissues around brackets could simulate a lifting effect on the wrinkles of the lower third of the face and thus invalidate the judgment of evaluators.

The anti-aging additional effect demonstrated, even if modest when compared with the results of aesthetic medicine procedures, could be partially considered by the clinicians, who nowadays are forced to face with ever increasing aesthetic demands without surgical and invasive procedures or procedures that provide the use of anesthesia and needles that have always been subjectively considered painful and looked with suspicious eyes in all medical fields (12).

## Conclusions

The properties and functional capacity of dental aligners may influence patients’ and aesthetics experts’ perception of facial beauty causing an additional anti-aging process.

In addition, the effect of wearing clear aligners can be interpreted as a complex and multiple phenomenon involving blood vessels, cells, growth factors, derma and muscle.

Such phenomenon has lead to a positive aesthetic impact of the face even if its results should be considered partial and interpreted with caution because of the need of further research.

## References

[1] Mohindra NK, Bulman JS (2002). The effect of increasing vertical dimension of occlusion on facial aesthetics. Br Dent J.

[2] Carruthers A, Carruthers J (2010). A validated facial grading scale: the future of facial ageing measurements tools?. J Cosmet Laser Ther.

[3] Day DJ, Littler CM, Swift RW, Gottlieb S (2004). The wrinkle severity rating scale: A validation study. Am J Clin Dermatol.

[4] Pelo S, Saponaro G, Patini R, Staderini E, Giordano A, Gasparini G (2017). Risks in surgery-first orthognathic approach: complications of segmental osteotomies of the jaws. A systematic review. Eur Rev Med Pharmacol Sci.

[5] Pelo S, Gasparini G, Garagiola U, Cordaro M, Di Nardo F, Staderini E (2017). Surgery-first orthognathic approach vs traditional orthognathic approach: Oral health-related quality of life assessed with 2 questionnaires. Am J Orthod Dentofacial Orthop.

[6] Nanda RS, Ghosh J (1995). Facial soft tissue harmony and growth in orthodontic treatment. Semin Orthod.

[7] Rossini G, Parrini S, Castroflorio T, Deregibus A, Debernardi CL (2015). Efficacy of clear aligners in controlling orthodontic tooth movement: a systematic review. Angle Orthod.

[8] Freesmeyer WB, Fussnegger MR, Ahlers MO (2005). Diagnostic and therapeutic-restorative procedures for masticatory dysfunctions. GMS Curr Top Otorhinolaryngol Head Neck Surg.

[9] Patini R, Arrica M, Di Stasio E, Gallenzi P, Cordaro M (2016). The use of magnetic resonance imaging in the evaluation of upper airway structures in paediatric obstructive sleep apnoea syndrome: a systematic review and meta-analysis. Dentomaxillofac Radiol.

[10] Okeson JP, Moody PM, Kemper JT, Calhoun TC (1983). Evaluation of occlusal splint therapy. J Craniomandibular Pract.

[11] Jones DB, Nolte H, Scholubbers JG, Turner E, Veltel D (1991). Biochemical signal transduction of mechanical strain in osteoblast-like cells. Biomaterials.

[12] Patini R, Coviello V, Raffaelli L, Manicone PF, Dehkhargani SZ, Verdugo F (2012). Subjective pain response to two anesthetic systems in dental surgery: traditional syringe vs. a computer controlled delivery system. J Biol Regul Homeost Agents.

